# Informal caregiving, time use and experienced wellbeing

**DOI:** 10.1002/hec.4624

**Published:** 2022-10-27

**Authors:** Sean Urwin, Yiu‐Shing Lau, Gunn Grande, Matt Sutton

**Affiliations:** ^1^ Health Organisation Policy and Economics Group School of Health Sciences University of Manchester Manchester UK; ^2^ Division of Nursing Midwifery and Social Work Manchester Academic Health Science Centre University of Manchester Manchester UK; ^3^ Melbourne Institute; Applied Economic and Social Research University of Melbourne Melbourne Australia

**Keywords:** informal care, time diary, time use, wellbeing

## Abstract

Informal carers report lower evaluative wellbeing than non‐carers. In contrast to this literature and our own analysis of evaluative wellbeing, we find carers have a small but higher level of experienced wellbeing than non‐carers do. To investigate why, we use decomposition analysis which separates explanatory factors into how time is used and how those uses of time are experienced. We analyze activities and associated experienced wellbeing measured in ten‐minute intervals over two days by 4817 adults from the 2014/15 UK Time Use Survey. We use entropy balancing to compare carers with a re‐weighted counterfactual non‐carer group and then apply Oaxaca‐Blinder decomposition. The experienced wellbeing gap of 0.066 is the net result of several substantial competing effects of time use. Carers experienced wellbeing would be higher by 0.188 if they had the same patterns and returns to time use as non‐carers which is driven by sleep, time stress and alternative characteristics of time use. However, leisure and non‐market activities serve to dampen this increase in experienced wellbeing. Initiatives to improve and assess carer wellbeing should pay close attention to how carers spend their time.

## 1 INTRODUCTION

One particular group of society that may incur reductions in wellbeing are informal carers. Informal carers are those who provide unpaid help to family and friends in need of assistance with daily activities. They make up a substantial proportion of populations worldwide. In 18 OECD countries in 2015, it was estimated that 10% of the population over 50 years old had some form of caregiving responsibility (Colombo et al., 2011). Given how widespread caregiving is, the effect of this care responsibility on wellbeing should be explored in detail.

Much of the health‐related literature on informal caregiving focuses on mental health (Bauer & Sousa‐Poza, 2015; Bom et al., 2019; Coe & Van Houtven, 2009; de Zwart et al., 2017; Do et al., 2015; Schmitz & Westphal, 2015) and, to a lesser extent, life satisfaction (Chen et al., 2019; Leigh, 2010; van den Berg et al., 2014; van den Berg & Ferrer‐I‐Carbonell, 2007). In general, caregivers have lower mental health and life satisfaction relative to non‐carers (see Bauer & Sousa‐Poza, 2015 and Bom et al., 2019 for reviews). However, these evaluative life satisfaction measures consider only one limited aspect of wellbeing, involving respondents comparing themselves to some societal standard (Kahneman & Krueger, 2006).

Wellbeing is a multifaceted concept consisting of many aspects. These can be categorized into three parts: evaluative wellbeing, which involves judgments of life satisfaction; experienced or hedonic wellbeing, which reflects a combination of positive or negative emotions/states; and eudaimonic wellbeing, which refers to a sense of meaning or purpose in life (Stone, 2013). It is unclear whether and by how much carers experienced wellbeing differs to non‐carers given previous evidence on evaluative wellbeing. Experienced wellbeing is closely tied to what activities an individual performs over the measurement period. This is particularly relevant in the case of caregiving as the type and duration of certain activities may not be similar to non‐carers. Therefore, it is appropriate to understand whether and how the experienced wellbeing of caregivers is affected by how a carer uses their time (referred to as time‐composition) or the returns to certain uses of time (referred to as saddening effects).

Experienced wellbeing measures can be further differentiated based on the method used to collect this information and are sometimes referred to as emotional or affective wellbeing. A common method in household surveys of measuring experienced wellbeing is a single‐item indicator relating to the feeling of an emotion such as happiness, worthwhileness or anxiousness in the previous or same day. A more involved measure consists of a combination of a time diary (detailing what respondents are doing) and the strength of various emotions at points in a day (known as experience sampling methods). This is referred to as the Daily Reconstruction Method developed by Kahneman et al. (2004). To gain a more complete picture of wellbeing, experienced wellbeing measures can complement the more commonly used evaluative measures (Dolan et al., 2017). This is relevant in the informal care literature where analysis using mental health and evaluative wellbeing measures are more commonplace (Bauer & Sousa‐Poza, 2015; Bom et al., 2019).

The time diary method of obtaining experienced wellbeing within economics has been used predominantly to explore differences in wellbeing by employment status (Hoang & Knabe, 2021; Knabe et al., 2010; Krueger & Mueller, 2012). The literature on differentials in experienced wellbeing decomposes these gaps between two effects—a “time‐composition” and “saddening” component (Flores et al., 2015, 2020; Hoang & Knabe, 2021; Knabe et al., 2010). Decomposing experienced wellbeing in this way was first explored by Knabe et al. (2010) who analyzed differences between the employed and unemployed. They identified the time‐composition effect by assuming the employed experience wellbeing in the same way as the unemployed but allowed time use to differ. For the saddening effect, they assumed that the employed had the same time use as the unemployed but the level of experienced wellbeing for the same activities was different. This thought‐experiment was further developed by Flores et al. (2015) across different activities and used to compare those with and without disabilities. More recently these decompositions in terms of “saddening” and “time‐composition” effects have been applied to experienced wellbeing differences by gender (Flores et al., 2020).

We are the first to analyze differences in daily experienced wellbeing by caregiving status using a time diary. Only one study, by Freedman et al. (2019), has analyzed daily experienced wellbeing between carers with a time diary method. They analyzed 511 time diaries from carers to understand how the amount of caregiving provided varied throughout the course of a day and the predictors of wellbeing differences within this carer group, but did not have a non‐carer comparator group.

We have two aims. The first is to explore if there are differences in daily experienced wellbeing by caregiving status. We compare this difference to that found with other evaluative wellbeing measures. To investigate why the daily experienced gap is of a particular size, our second aim decomposes the difference by uses of and experiences of time. We refer to these effects as “time‐composition” and “saddening” effects, respectively.

We make three contributions to the literature on informal care and wellbeing. These are made possible due to the rich time use and wellbeing data available from the 2014/15 UK Time Use Survey (Sullivan & Gershuny, 2020). First, we analyze a rarely explored aspect of wellbeing that has mainly been considered across employment statuses, but also provide measures of evaluative and eudaimonic wellbeing for comparison. Second, we consider three innovative aspects of time use (which are joint production, the fragmentation of time and subjective feelings of time stress) as potential determinants of daily experienced wellbeing. Previous research in this area has only identified the degree to which carers jointly produce activities and has not considered its implication for wellbeing. Third, we use the Oaxaca‐Blinder decomposition method (Blinder, 1973; Oaxaca, 1973) to identify two key components that may drive differences in daily experienced wellbeing: a time‐composition effect and a “saddening effect”. The time‐composition component relates to carers spending different amounts of time on more enjoyable activities relative to non‐carers. The time effects component relates to whether carers experience different wellbeing returns for the same activities as non‐carers. We address the previously identified issue of common support with the Oaxaca decomposition method by combining the method with entropy balancing (Hainmueller, 2012).

## 2 DATA

We use the 2014/15 UK Time Use Survey (Sullivan & Gershuny, 2020) to explore daily experienced wellbeing differences by caregiving status. This is a nationally representative survey from all countries of the United Kingdom. All respondents in a surveyed household are asked to first complete an individual questionnaire and then randomly assigned 2 days to complete a time diary: on a week day and a weekend day. The survey contains 16,533 diary‐days, from 8274 individuals residing in 4230 private households. Data collection started in January 2014 and ended in December 2015 with the aim of allocating diaries that are evenly distributed throughout the year to account for seasonality.

The time diary divides the day into 144 ten‐minute segments across a 24‐h period from 4 to 4 a.m. the subsequent day. Upon completion, the diary is sent to coders who then apply the 276 pre‐specified activity codes to convert text answers to numerical ones (Sullivan & Gershuny, 2020). The coding structure of the time diaries is hierarchical. The least granular are one‐digit activity codes (of which there are 11) and the most granular are four‐digit activity codes.

A feature of the UK Time Use Survey in 2014/15 was the addition of an experienced wellbeing component for the first time. Each episode could be rated based on the question:

How much did you enjoy this time?

Coded on a 1 (not at all) to 7 (very much) scale. An episode of activity is defined as one uninterrupted primary activity which ends when a different activity is performed. This wellbeing component is similar to the Daily Reconstruction Method developed by Kahneman et al. (2004).

### 2.1 Wellbeing outcomes

We use two sets of wellbeing outcomes. The first set contains five single‐item measures of either evaluative, experienced or eudaimonic wellbeing from the individual questionnaire:

(i) Life satisfaction overall, worded as:
*“How dissatisfied or satisfied would you say you are with your life overall?”* measured on a 1 (completely dissatisfied) to 7 (completely satisfied) scale.
(ii) Satisfaction with life nowadays, worded as:
*“Overall how satisfied are you with your life nowadays?”* measured on a 0 (not at all satisfied) to 10 (completely satisfied) scale.
(iii) Level of happiness, worded as:
*“Overall, how happy did you feel yesterday?”* measured on a 0 (not happy at all) to 10 (completely happy) scale.
(iv) Level of worthwhileness, worded as:
*“Overall, to what extent do you feel that the things you do in your life are worthwhile?”* measured on a 0 (not at all worthwhile) to 10 (completely worthwhile) scale.
(v) Level of anxiety, worded as:
*“On a scale where naught is ‘not at all anxious’ and 10 is ‘completely anxious’, overall, how anxious did you feel yesterday?”*  This measure is inverse coded to bring it in line with measures (i) to (iv). Therefore, higher values represent lower levels of anxiousness.

Measures (ii)–(v) are four measures described as personal wellbeing that are used by the ONS (Office for National Statistics, 2020). The measures can be classified where: (i) is an evaluative wellbeing measure; (iv) a eudaimonic wellbeing measure; and the remainder ([ii], [iii], and [v]) are single‐item experienced wellbeing measures.

The second wellbeing measure set is experienced wellbeing derived from the time diary. We construct an average daily measure from each time diary that aggregates all experienced wellbeing scores regardless of the activity undertaken to one figure:

$${DWB}_{j}=\sum_{n=1}^{N_{j}}\frac{t_{nj}}{T}w_{nj}$$

Where $N_{j}$ is the number of episodes for diary *j*, $t_{nj}$ is the total time spent on episode *n*, T is the total time recorded for the diary day, and $w_{nj}$ the wellbeing score for each episode which is used to create the daily experienced wellbeing score denoted ${DWB}_{j}$. Hoang and Knabe (2021) have used this method to aggregate experienced wellbeing to a daily level.

### 2.2 Informal care

Respondents are asked in the individual questionnaire to declare if they give informal help to a co‐residing household member:

Is there anyone living with you who is sick, disabled or elderly whom you look after or give special help to, other than in a professional capacity?

We classify those who answer yes to this question as an informal carer.

### 2.3 Time use variables and other covariates

Our primary variables of interest relate to objective and subjective measures of time use. The objective measures are proportions of time spent on each of six activities across the diary day. These activity groups have been used previously in the literature on time use (Gimenez‐Nadal & Sevilla, 2012) which are sleep, personal care, market work, non‐market work, leisure, and miscellaneous activities. Personal care includes activities such as eating. Market work relates to employment or education activities. Non‐market work includes household upkeep, childcare and help to other households. Leisure includes social life, physical exercise, hobbies and games, TV and video activities and finally miscellaneous relates to other unspecified time. We assign associated travel times to each respective activity. We provide detail of each (two‐digit) activity that is contained within our six time use categories in appendix Table A1. To understand if these activity groups may mask the effects of activities within each category we also use a more granular grouping of 11 activities listed in appendix Table A2.

We consider three additional characteristics of time use which are: (i) the proportion of the day spent jointly producing activity which is sometimes referred to as multitasking; (ii) the total number of episodes throughout the day with each episode defined as a continuous period on one primary activity; and (iii) a measure of “time stress”. A respondent jointly produces if they record a secondary activity alongside a primary activity in the diary. The subjective measure of “time stress” from the individual questionnaire is worded as:

In general, how rushed do you normally feel?

A respondent can answer: “always”; “sometimes” or “never”.

We include characteristics based on the literature that has either examined the determinants of caregiving (Mentzakis et al., 2009), has modeled selection into caregiving with matching methods (Bom & Stöckel, 2021; Schmitz & Westphal, 2017) or instrumented for informal care with the first stage of two‐stage least squares (Bolin et al., 2008; Bonsang, 2009; Urwin et al., 2019; Van Houtven & Norton, 2004). We include the following covariates from the individual questionnaire: gender; age; marital status; whether the respondent lives with their partner; the highest education qualification; home ownership; whether the respondent has a long‐term health condition; the number of adults and children in the household; household composition type and region. We provide additional details of these variables in appendix Table A3. From the diary we include indicators for the day of the week and the season of the year during which the diary was completed.

## 3 METHODS

### 3.1 Decomposition of the wellbeing gap

We decompose daily experienced wellbeing differences at the average by caregiving status. We use the Oaxaca Blinder decomposition method (Blinder, 1973; Oaxaca, 1973) which begins with linear regressions stratified by caregiving status:

$$W_{iN}={{\hat{\beta }}_{N}X}_{iN}-e_{iN}$$

$$W_{iC}={{\hat{\beta }}_{C}X}_{iC}-e_{iC}$$

Where $W_{ic}$, $X_{ic}$, ${\hat{\beta }}_{ic},e_{ic}$ denote the wellbeing, all time use variables, coefficients and the error term for individual i further denoted with subscript C for the carer group and subscript N for the non‐carer group. The zero expected mean assumption for the error terms where $E\left(e_{ic}\right)=0$ and $E\left(e_{iN}\right)=0$ allows the difference between the two groups to be evaluated as:

$$\begin{array}{lll} {\bar{W}}_{N}-{\bar{W}}_{C} & = & E\left({\bar{W}}_{N}\right)-E\left({\bar{W}}_{C}\right)\\ & = & {{\bar{X}}_{N}}^{\prime }{\hat{\beta }}_{N}-{{\bar{X}}_{C}}^{\prime }{\hat{\beta }}_{C} \end{array}$$

This is the starting point for many variants of the Oaxaca Blinder decomposition which involve rearranging this equation to decompose mean differences into several components. In this paper, we use the threefold decomposition given in Equation (1):

(1) $${\bar{W}}_{N}-{\bar{W}}_{C}={\left({\bar{X}}_{N}-{\bar{X}}_{C}\right)}^{\prime }{\hat{\beta }}_{N}-{\bar{X}}_{N}\left({\hat{\beta }}_{N}-{\hat{\beta }}_{C}\right)-{\left({\bar{X}}_{N}-{\bar{X}}_{C}\right)}^{\prime }\left({\hat{\beta }}_{N}-{\hat{\beta }}_{C}\right)$$

The threefold decomposition contains three distinct parts: the explained, unexplained and interaction effects. We refer to each part hereafter as the time‐composition effect, time effect and interaction effect, respectively. The first part is the group differences in the levels of the explanatory variables $\left({\bar{X}}_{N}-{\bar{X}}_{C}\right)$ weighted by the coefficients of non‐carers $\left({\hat{\beta }}_{N}\right)$. It describes how much of the gap in daily experienced wellbeing is due to the differences in time use. This component is quantifying what would happen to daily experienced wellbeing if carers spent the same *level* of time on various activities as non‐carers.

The second part is the differences in coefficients $\left({\hat{\beta }}_{N}-{\hat{\beta }}_{C}\right)$; weighted by the explanatory variables $\left({\bar{X}}_{N}\right)$ of non‐carers. This component is quantifying what would happen to daily experienced wellbeing if carers had the same *effects* of time spent on various activities as non‐carers.

The final part ${\left({\bar{X}}_{N}-{\bar{X}}_{C}\right)}^{\prime }\left({\hat{\beta }}_{N}-{\hat{\beta }}_{C}\right)$ represents the proportion of the wellbeing difference i.e., due to the interaction of the time‐composition and time effect components. The interaction effect accounts for simultaneous differences in the time‐composition and time effects between carers and non‐carers. In other words, non‐carers’ coefficients may be larger than carers' coefficients for certain uses of time which non‐carers have lower levels than carers. Research using the Oaxaca Blinder decomposition often treats the interaction component as being part of the time effects component, using twofold decomposition (Jann, 2008). However, we include this component because it is unclear a‐priori whether it will account for a substantial part of the difference.

We treat non‐carers as the benchmark group given that they are the larger of the two groups. Therefore, the time‐composition effect can be described as the expected change in carers daily experienced wellbeing if they had the same time use as non‐carers. Similarly, the time effect is the expected change in carers daily experienced wellbeing if they had the same coefficients as non‐carers. We present the results for each time use variable as the contribution of certain variables may be larger than the experienced daily wellbeing gap and therefore obscured if only summaries of each component are presented.

A common issue with the Oaxaca‐Blinder decomposition is that the contribution of categorical variables can depend upon which base category is chosen (Jann, 2008). For the proportion variables relating to time use, we use market work as the base category, because mean daily experienced wellbeing for market work is the lowest for non‐carers and therefore the coefficients for all other uses of time represent positive wellbeing returns. For time stress, we omit the category for those who never feel time stressed. This allows more comparable interpretation to that for joint production and the total number of episodes, as increases in all these aspects represent worse daily experienced wellbeing.

An advantage with the Oaxaca‐Blinder decomposition is that it enables an understanding of how much the daily experienced wellbeing of carers would increase or decrease if: (i) they spend the same amount of time as non‐carers on relatively more (or less) enjoyable activities or (ii) they receive the same wellbeing returns as non‐carers on for each activity.

For completeness of the method we report standard errors clustered by the primary sampling unit which consists of the postcode sector in Great Britain and wards in Northern Ireland. We interpret the standard errors with caution. Etezady et al. (2021) state that caution around their interpretation is because a component with low statistical significance cannot be equated to equal zero otherwise all other terms in the decomposition would not equal the total gap.

### 3.2 Entropy weighting

Another issue with the Oaxaca‐Blinder decomposition is the “out‐of‐support” assumption when comparing differences between two groups (Ñopo, 2008). Comparison of daily experienced wellbeing by caregiving status may only be relevant amongst carers and non‐carers with similar characteristics. Ñopo (2008) provides a solution that combines the Oaxaca‐Blinder decomposition with a matching technique using the male‐female wage gap as an example. Specifically, Ñopo (2008) uses one‐to‐one exact matching with replacement with the aim to construct a counterfactual group.

In cases with many covariates the “curse of dimensionality” prohibits exact matching. As an alternative, in the present study, we apply entropy weights using the “ebalance” command in STATA (Hainmueller, 2012). These weights are applied to the sample so that balance is achieved between carer and non‐carer groups in terms of various moments of the covariates. We balance on the mean for indicator variables and the mean, variance and skewness for continuous covariates. Entropy weighting has the advantage of efficiency over the more commonly used propensity score methods that often removes observations that cannot be assigned to a treated observation (Hainmueller, 2012).

### 3.3 Sample restrictions

There are 9627 time diaries from 4817 individuals who are at least 18 years old in the 2014/15 UK Time Use Survey with a wellbeing component. Approximately 85% of adult respondents had completed a wellbeing version of the time diary. For categorical covariates with missing values, we create an extra category to preserve the sample size.

It is unclear how much of a time diary should be complete, with respect to wellbeing information, before it can be judged as “useable”. Guidance for the 2000/01 UK Time Use Survey, the predecessor to the 2014/15 UK Time Use Survey, classed “good quality” as having no more than 90 min of incomplete time information, but this did not include an experienced wellbeing component. We use this marker of quality in our main analysis, requiring diaries to have no more than 90 min of incomplete time and wellbeing information. We further remove diaries where more than 80% of the day was spent sleeping. These extreme values are removed based on visual inspection as sleep can cover a considerable proportion of the day and this may have substantial effects on estimated coefficients from the returns to sleeping. We remove four non‐carer observations as these have an entropy weight of zero. These restrictions reduce the sample to 6151 diaries from 3513 individuals. From the initial sample this is a reduction of 36% of dairies and 27% of individuals.

### 3.4 Robustness checks

To consider the importance of these sample restrictions, we present as robustness checks the results from decomposition analysis across two additional samples with different restrictions placed on them. These are diaries with full completeness of time and wellbeing information and diaries with no restrictions on the completeness of time and wellbeing information.

We present the difference in daily experienced wellbeing between carers and non‐carers for each 10‐min relaxation of the time use and wellbeing restriction among diaries with no sleep time outliers. This is to understand if the sample restriction we impose results in an atypical difference in wellbeing. To complement this analysis, we present the sample characteristics by caregiving status at both extremes of the completeness restrictions.

We further present decomposition analysis which excludes covariates from entropy weighting that could be considered “bad controls” as they may impact on informal care provision. This analysis excludes marital status, living with partner and long‐standing illness variables used in the estimation of entropy weights.

We attempt to account for selection into caregiving using all the relevant covariates available in the UK Time Use Survey and through the use of entropy balancing. There may still be unobservable factors that we are unable to account for such as health or attachment to the labor market. These issues can be addressed to a greater extend with panel data, for instance, among studies that aim to identify the effect of informal care on mental health, physical health and evaluative wellbeing (see, e.g., Bom and Stöckel (2021), Leigh (2010); Schmitz and Westphal (2017) and van den Berg et al. (2014)). We therefore interpret our results as correlations. However, to show the sensitivity of our results to the matching method we present a decomposition in the appendix using inverse probability of treatment weights.

## 4 RESULTS

### 4.1 Entropy weighting on covariates

Carers are older, more likely to be female, married/cohabiting, UK born and reside in households with more adults compared to non‐carers. The application of entropy weights results in balance within one percentage point for the mean of binary variables listed in Table 1 by caregiving status. Summary statistics of the covariates for the sample with no incomplete slots and for a sample with no restrictions are shown in appendix Table A4. The characteristics of the non‐carer and carers groups are similar for the full range of sample restrictions.

**TABLE 1:** Sample balance with entropy weights

	Carer diaries (*N* = 361)		Non‐carer diaries (*N* = 5790)			
			Pre‐balance		Post‐balance	
Covariate	Mean	Variance	Mean	Variance	Mean	Variance
Individual level covariates						
Female	0.58	0.24	0.51	0.25	0.58	0.24
Age (years)	53.21	254.88	48.68	335.54	53.18	254.77
Marital status: Married/cohabiting	0.80	0.16	0.58	0.24	0.80	0.16
Marital status: Divorced/widowed	0.09	0.08	0.16	0.13	0.09	0.08
Marital status: Missing	0.00	0.00	0.08	0.07	0.00	0.00
Lives with spouse	0.80	0.16	0.65	0.23	0.80	0.16
Education: Higher secondary qualification	0.15	0.13	0.18	0.15	0.15	0.13
Education: Secondary qualification	0.29	0.21	0.23	0.18	0.29	0.21
Education: Other qualification	0.16	0.13	0.11	0.10	0.16	0.13
Education: Missing	0.06	0.06	0.10	0.09	0.06	0.06
Tenure: Owns home	0.64	0.23	0.71	0.21	0.64	0.23
Born: UK	0.91	0.08	0.80	0.16	0.91	0.08
Born: Missing	0.00	0.00	0.08	0.07	0.00	0.00
Long standing health condition: Yes	0.48	0.25	0.33	0.22	0.48	0.25
Long standing health condition: Missing	0.00	0.00	0.08	0.07	0.00	0.00
# of Adults in HH	2.66	1.26	2.27	1.18	2.66	1.36
# aged 11–15 years old in HH	0.21	0.23	0.16	0.19	0.21	0.25
# aged 5–10 years old in HH	0.29	0.39	0.18	0.24	0.29	0.39
# aged 0–4 years old in HH	0.12	0.16	0.19	0.25	0.12	0.16
Region: London/south east	0.25	0.19	0.32	0.22	0.25	0.19
Region: Wales	0.01	0.01	0.01	0.01	0.01	0.01
Region: Scotland	0.01	0.01	0.00	0.00	0.01	0.01
HH type: Married/Cohab with children	0.14	0.12	0.19	0.15	0.14	0.12
HH type: Married/Cohab no children	0.36	0.23	0.30	0.21	0.36	0.23
HH type: Single parent with children	0.04	0.04	0.03	0.03	0.04	0.04
HH type: Single parent no children	0.04	0.04	0.03	0.03	0.04	0.04
HH type: Unclassified – married/cohab	0.22	0.17	0.16	0.13	0.22	0.17
HH type: Unclassified – single parent	0.14	0.12	0.08	0.07	0.14	0.12
HH type: Other households	0.06	0.05	0.04	0.04	0.06	0.05
Diary level covariates						
Season: Spring	0.25	0.19	0.22	0.17	0.25	0.19
Season: Summer	0.24	0.18	0.16	0.13	0.24	0.18
Season: Autumn	0.25	0.19	0.33	0.22	0.25	0.19
Day of the week: Tuesday	0.11	0.10	0.09	0.09	0.11	0.10
Day of the week: Wednesday	0.11	0.09	0.10	0.09	0.11	0.09
Day of the week: Thursday	0.10	0.09	0.09	0.08	0.10	0.09
Day of the week: Friday	0.10	0.09	0.10	0.09	0.10	0.09
Day of the week: Saturday	0.07	0.06	0.11	0.10	0.07	0.06
Day of the week: Sunday	0.27	0.20	0.25	0.19	0.27	0.20

Abbreviations: cohab, cohabiting; HH, Household.

### 4.2 Wellbeing and time use summary statistics

**FIGURE 1:**
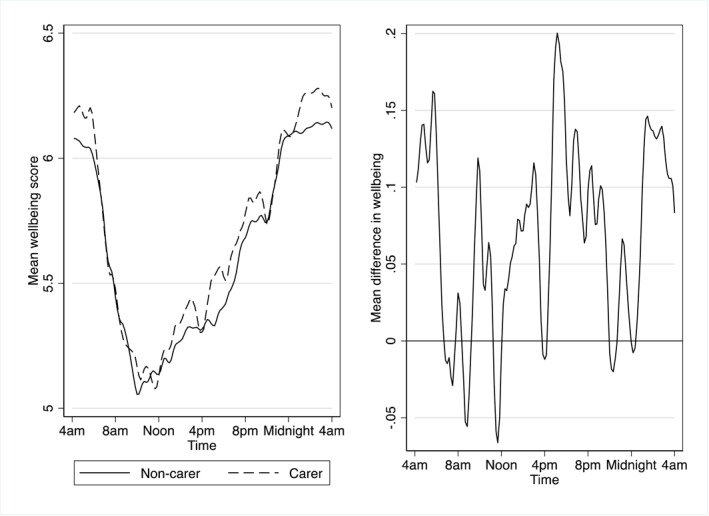
Average experienced wellbeing across a diary day for carers and non‐carers. Entropy weights are applied. The left‐hand side figure is a local linear smooth plot. The right‐hand side figure is the difference in the smoothened plot which is calculated as carer minus non‐carer wellbeing

Figure 1 presents daily experienced wellbeing scores within the diary day by caregiving status regardless of the activity type. Daily experienced wellbeing for both carers and non‐carers becomes worse as the day progresses until noon and then increases thereafter. The majority of differences in daily experienced wellbeing range between 0.2 and −0.05 units. Carers have higher experienced wellbeing scores, on average, from midnight to 6a.m. indicated by the positive difference in the right‐hand side graph in Figure 1. Some of the carer experienced wellbeing deficit may be because sleep is more enjoyable and carers spent less time on this activity between 6a.m. and Noon. These figures demonstrate that the differential between carers and non‐carers varies throughout the day.

Figure A5 shown in the appendix plots daily experienced wellbeing and the proportion of time spent on each activity. On visual inspection the linear trends with entropy weights applied appear similar for carers and non‐carers across all non‐miscellaneous activities.

We apply diary level entropy weights from the main analysis sample to all variables including the single‐item wellbeing measures in Table 2. Carers have an average daily experienced wellbeing score of 5.76 whereas for non‐carers this is lower at 5.73 (Table 2). The application of entropy weights decreases non‐carers average daily experienced wellbeing to 5.69 resulting in a 0.066 difference compared to carers. To put this difference into context it is approximately 25% of the 0.250 difference between the average daily wellbeing score of weekday and weekend diaries.

**TABLE 2:** Summary statistics of wellbeing outcomes and time use

	Carers		Non‐carers			Difference	
	*N*	Mean (SD)	*N*	Unweighted mean (SD)	Weighted mean (SD)	Unweighted (SE)	Weighted (SE)
Daily measure of experienced wellbeing							
Daily average	361	5.76 (0.75)	5786	5.73 (0.85)	5.69 (0.87)	0.027 (0.052)	0.066 (0.055)
Sleep	361	6.29 (0.98)	5786	6.22 (1.05)	6.19 (1.07)	0.072 (0.066)	0.098 (0.069)
Personal care	360	5.73 (0.92)	5763	5.67 (0.98)	5.63 (0.98)	0.058 (0.060)	0.101 (0.065)
Market work	161	4.50 (1.28)	2363	4.70 (1.31)	4.65 (1.31)	−0.196 (0.122)	−0.149 (0.129)
Non‐market work	350	5.30 (1.09)	5427	5.15 (1.20)	5.09 (1.21)	0.154** (0.076)	0.216*** (0.080)
Leisure	360	5.94 (0.82)	5725	5.92 (0.91)	5.91 (0.91)	0.019 (0.055)	0.029 (0.057)
Miscellaneous	135	5.60 (1.23)	1951	5.28 (1.50)	5.25 (1.53)	0.322** (0.129)	0.350** (0.141)
Single‐item wellbeing measures							
Overall life satisfaction *(evaluative wellbeing)*	166	5.13 (1.48)	2338	5.51 (1.32)	5.44 (1.39)	−0.383** (0.175)	−0.316* (0.185)
Life satisfaction nowadays *(experienced wellbeing)*	155	7.40 (1.89)	2148	7.47 (1.92)	7.50 (1.85)	−0.071 (0.222)	−0.097 (0.230)
Level of happiness *(experienced wellbeing)*	155	6.72 (2.57)	2149	7.27 (2.32)	7.18 (2.42)	−0.549** (0.247)	−0.457 (0.280)
Level of worthwhileness *(eudaimonic wellbeing)*	155	7.92 (1.81)	2146	7.67 (1.88)	7.70 (1.93)	0.244 (0.215)	0.214 (0.218)
Level of anxiousness *(experienced wellbeing)*	155	6.66 (3.18)	2149	7.04 (2.89)	7.10 (2.83)	−0.382 (0.336)	−0.446 (0.361)
Time use							
Sleep	361	35.83 (7.05)	5786	36.240 (7.96)	35.95 (8.11)	−0.406 (0.389)	−0.112 (0.416)
Personal care	361	11.11 (6.29)	5786	10.34 (5.60)	10.21 (5.52)	0.779* (0.445)	0.902* (0.465)
Market work	361	13.46 (18.06)	5786	12.21 (17.69)	12.42 (17.88)	1.240 (1.060)	1.034 (1.111)
Non‐market work	361	16.05 (12.00)	5786	15.91 (12.10)	16.16 (12.06)	0.143 (0.759)	−0.113 (0.789)
Leisure	361	22.45 (12.21)	5786	24.14 (13.12)	24.07 (13.49)	−1.702** (0.723)	−1.618** (0.742)
Miscellaneous	361	1.10 (2.98)	5786	1.16 (3.44)	1.20 (3.47)	−0.054 (0.169)	−0.093 (0.213)
Additional time use characteristics							
Proportion of the day joint producing	361	0.19 (0.16)	5786	0.19 (0.15)	0.18 (0.14)	0.002 (0.012)	0.009 (0.013)
Total number of episodes	361	35.11 (13.00)	5786	34.58 (13.02)	34.55 (13.17)	0.533 (0.879)	0.558 (0.950)
Time stress: % always	361	0.17 (0.38)	5786	0.13 (0.34)	0.14 (0.35)	0.036 (0.027)	0.024 (0.028)
Time stress: % sometimes	361	0.57 (0.50)	5786	0.46 (0.50)	0.48 (0.50)	0.105*** (0.035)	0.089** (0.038)
Time stress: % never	361	0.15 (0.36)	5786	0.18 (0.38)	0.19 (0.39)	−0.029 (0.026)	−0.039 (0.029)
Time stress: % Missing	361	0.11 (0.31)	5786	0.22 (0.42)	0.19 (0.39)	−0.113*** (0.025)	−0.074*** (0.024)

*Note*: Entropy weights from the main analysis are applied to all variables. An alternative way would be to produce new entropy weights for complete cases, for example, to the sub‐sample for each single item wellbeing measure.

*p < 0.1; **p < 0.05; ***p < 0.01.

Carers have a higher daily experienced wellbeing score compared to non‐carers, on average, for sleep, personal care, leisure and non‐market work. Carers have a 0.216 (*p* < 0.1) higher and a 0.149 (*p* > 0.1) lower daily experienced wellbeing score for non‐market and market work, respectively, which is similar to the gap between weekday and weekend diaries. Across all evaluative and single‐item experienced wellbeing measures carers report lower wellbeing with a difference compared to non‐carers of 0.457 (*p* > 0.1) for happiness and 0.316 (*p* < 0.1) for satisfaction with life overall. However, carers have a higher average level of worthwhileness than non‐carers by 0.214 (*p* > 0.1).

Carers spend less time on leisure by 1.62% (*p* < 0.05) of the day (equivalent to 23 min) which suggests a time‐composition effect. Both carers and non‐carers spend a similar proportion of the day jointly producing and have a total number of episodes at 34.58 and 35.11, respectively. A higher proportion of carers report feeling always or sometimes time stressed relative to non‐carers.

Table 3 presents the correlations between wellbeing measures. We find that daily experienced wellbeing is not strongly correlated with any of the evaluative measures among carers or non‐carers. There are stronger correlations between the various evaluative wellbeing measures for both carers and non‐carers.

**TABLE 3:** Correlation across experienced and evaluative wellbeing measures

		Wellbeing measure				
	Daily exp wellbeing	Overall life satisfaction	Satisfaction with life nowadays (N)	Happiness	Worthwhileness	Anxiety
Carers (N)						
Daily experienced wellbeing	1					
	(361)					
Overall life satisfaction	0.08	1				
	(166)	(166)				
Satisfaction with life nowadays	0.02	‐	1			
	(155)	‐	(155)			
Happiness	0.02	‐	0.39*	1		
	(155)	‐	(155)	(155)		
Worthwhileness	−0.01	‐	0.64*	0.31*	1	
	(155)	‐	(155)	(155)	(155)	
Anxiety	−0.01	‐	0.28*	0.65*	0.13	1
	(155)	‐	(155)	(155)	(155)	(155)
Non‐carers (N)						
Daily experienced wellbeing	1					
	(5786)					
Life satisfaction	0.03	1				
	(2338)	(2338)				
Satisfaction	0.05*	‐	1			
	(2148)	‐	(2148)			
Happiness	−0.05*	‐	0.45*	1		
	(2149)	‐	(2145)	(2149)		
Worthwhileness	0.002	‐	0.58*	0.35*	1	
	(2146)	‐	(2144)	(2144)	(2146)	
Anxiety	0.03	‐	0.26*	0.29*	0.29*	1
	(2149)	‐	(2145)	(2147)	(2144)	(2149)

*Note*: We report Pearson's correlation coefficient. Correlations are performed at a diary level with the application of entropy weights from the main analysis sample. Overall life satisfaction was asked to a different sub‐sample than the experienced and eudaimonic wellbeing measures. Sample sizes are in parentheses and *p < 0.1 indicates the statistical significance of the correlations.

### 4.3 Experienced wellbeing regressions

We present regressions with daily experienced wellbeing as the outcome variable stratified by caregiving status in Table 4. The direction of all the coefficients are the same for non‐carers and carers with the exception of time stress, but there are substantial differences in magnitudes. Carers have lower returns to sleep, as an extra 1% of the day spent on sleep (equivalent to 14.4 min), increases daily experienced wellbeing by 0.011 (*p* < 0.05) for carers and 0.013 (*p* < 0.01) for non‐carers. The magnitude on the leisure coefficient is larger for carers at 0.014 (*p* < 0.01) than non‐carers at 0.011 (*p* < 0.01). More time spent jointly producing and performing a higher number of episodes are related to a greater experienced wellbeing deficit for carers compared to non‐carers. Carers report a daily experienced wellbeing deficit from always being time stressed of −0.347 (*p* < 0.1) compared to those that never report time stress. The equivalent coefficient is close to zero at −0.001 (*p* > 0.1) for non‐carers.

**TABLE 4:** Regressions on daily experienced wellbeing stratified by caregiving status

	Carers	Non‐carers
Sleep	0.011**	0.013***
	(0.005)	(0.003)
Personal care	0.017**	0.020***
	(0.007)	(0.004)
Non‐market work	0.015***	0.011***
	(0.004)	(0.003)
Leisure	0.014***	0.011***
	(0.004)	(0.002)
Miscellaneous	0.014	0.001
	(0.011)	(0.010)
% day joint producing	−0.261	−0.034
	(0.283)	(0.226)
Total number of episodes	−0.007*	−0.006**
	(0.004)	(0.002)
Time stress: Always	−0.347*	−0.001
	(0.204)	(0.093)
Time stress: Sometimes	−0.118	0.033
	(0.147)	(0.081)
Time stress: Missing	−0.127	−0.079
	(0.199)	(0.135)
Constant	5.758***	5.692***
	(0.049)	(0.032)
Diaries	361	5786
Individuals	206	3304

*Note*: Diaries that report less than 80% of the day sleeping and with no more than 90 min of incomplete time use and wellbeing information are analyzed. The daily experienced wellbeing score is the average score across the day from a scale coded from 1 (“not at all”) to 7 (“very much”) where higher scores indicate better wellbeing. Entropy weights have been applied to each regression. The omitted time category and time stress category is market work and never, respectively. Non‐time related covariates included in the construction of entropy weights are: gender; age; marital status; whether the respondent lives with their partner; the highest education qualification; home ownership; whether the respondent has a long‐term health condition; the number of adults and children in the household; household composition type; region; diary day of the week; and diary season. Standard errors are clustered by the primary sampling unit in parenthesis.

*p < 0.1; **p < 0.05; ***p < 0.01.

Regressions from each of the two alternative samples are presented in Table A6. Sample restrictions on the carer sample have the most impact on the sleep coefficient which is 0.008 (*p* > 0.1) for the complete case sample and 0.016 (*p* < 0.001) for the no restriction sample. Carer time use coefficients for the no restrictions sample are closer to non‐carers than results from Table 4 and the complete case sample.

### 4.4 Decomposition with six time use activities

Positive contributions in Table 5 represent increases in carer daily experienced wellbeing if they had the same level of time use or time effects as non‐carers, serving to increase the positive experienced wellbeing gap. Carers daily experienced wellbeing would increase by 0.081 if the time they spent sleeping and the returns to sleep were as high as non‐carers. This change is substantial, broadly equivalent to the wellbeing difference by longstanding illness status. If carers had a lower proportion of those in higher feelings of time stress and exhibited a smaller daily experienced wellbeing change from increasing time stress as non‐carers their wellbeing would increase by 0.142 (= 0.084 + 0.058). Carers daily experienced wellbeing would increase by 0.076 (= 0.043 + 0.033) if they spent a smaller amount of time jointly producing and had fewer episodes as well as less negative returns to these characteristics in line with non‐carers. The time effects rather than the time‐composition components are the dominant components among the positive contributors. However, personal care has the only negative time‐composition effect among overall positive time use contributors. This contrast is because carers spend more time on this more enjoyable activity relative to non‐carers.

**TABLE 5:** Oaxaca‐Blinder decomposition of the experienced wellbeing gap between carers and non‐carers

Variable	Time‐composition (SD)	Time effect (SD)	Interaction (SD)	Total (SD)
Time stress: Always	0.011	0.086	−0.013	0.084
	(0.102)	(0.649)	(0.102)	
Sleep	0.001	0.080	0.000	0.081
	(0.007)	(1.588)	(0.005)	
Time stress: Sometimes	0.008	0.058	−0.008	0.058
	(0.038)	(0.258)	(0.038)	
% day jointly produced	0.002	0.043	−0.002	0.043
	(0.022)	(0.450)	(0.022)	
Total number of episodes	0.004	0.029	−0.000	0.033
	(0.019)	(1.155)	(0.018)	
Personal care	−0.016	0.024	−0.002	0.006
	(0.052)	(0.641)	(0.052)	
Time stress: Missing	−0.009	0.005	0.004	0.000
	(0.110)	(0.164)	(0.110)	
Miscellaneous	0.001	−0.014	−0.001	−0.014
	(0.009)	(0.102)	(0.009)	
Leisure	0.022	−0.064	−0.005	−0.047
	(0.049)	(0.668)	(0.048)	
Non‐market	0.002	−0.057	−0.000	−0.055
	(0.013)	(0.576)	(0.005)	
Constant		−0.254		−0.254
		(2.675)		
Total	0.026	−0.064	−0.029	−0.066
	(0.121)	(0.413)	(0.121)	(0.413)

*Note*: The total mean difference is calculated as non‐carer minus carer diaries in line with Equation (1). Diaries that report less than 80% of the day sleeping and with no more than 90 min of incomplete time use and wellbeing information are analyzed. The decomposition is applied to a sample of 5786 non‐carer diaries from 3304 non‐carer individuals and 361 carer diaries from 206 carers. Non‐carers are the reference group. The daily experienced wellbeing score is the average score across the day from a scale coded from 1 (“not at all”) to 7 (“very much”) where higher scores indicate better wellbeing. Entropy weights have been applied to the decomposition. The omitted time category and time stress category is market work and always, respectively. Non‐time related covariates included in the construction of entropy weights are: gender; age; marital status; whether the respondent lives with their partner; the highest education qualification; home ownership; whether the respondent has a long‐term health condition; the number of adults and children in the household; household composition type; region; diary day of the week; and diary season. Standard errors clustered at the primary sampling unit in parentheses.

*p < 0.1; **p < 0.05; ***p < 0.01.

Negative contributions represent decreases in daily experienced wellbeing if carers had the same time‐composition and time effects as non‐carers. The largest contributor in absolute terms is the constant. This highlights the membership effect or positive aspects to day‐to‐day life from becoming a carer. In other words, it is the unexplained daily experienced wellbeing difference from becoming a carer of 0.254 aside from time use or differences in observable characteristics. If carers had lower daily experienced wellbeing returns to leisure as non‐carers do then daily experienced wellbeing would decrease by 0.064. In contrast, carers spend less time on leisure, but if they increased this amount of time to that of non‐carers, daily experienced wellbeing would increase by 0.022 given that leisure ranks as one of the most enjoyable activities. Although the net effect of leisure is negative by 0.047 as the time effect dominates. There is a similar contrast with non‐market work having a small but positive time‐composition effect.

The total of the time‐composition effects show that carer daily experienced wellbeing would increase by 0.026 if they had the same time use and level of time stress as non‐carers. If carers had the same returns to time use and time stress as non‐carers their experienced wellbeing would decrease by 0.064 showing that the time effect dominates. Exclusion of the constant term would result in a positive total time effects component of 0.19. This emphasizes that carers daily experienced wellbeing would be even higher overall if their level of and returns to time use and stress were closer to that of non‐carers.

We present the full Oaxaca‐Blinder decomposition for the complete case sample in Table A7 and the no restrictions sample in Table A8. The magnitude of results in Table 5 are comparable to the decomposition estimated on the complete case sample in Table A7, despite carers having lower daily experienced wellbeing relative to non‐carers for this sample. The difference in daily experienced wellbeing is identical for the sample with no restriction on completeness to our main sample at −0.066. Decomposition results for the no restrictions sample in Table A8 contrast with our main analysis sample results as the constant is closer to zero and carers have a higher daily experienced wellbeing returns to sleep than non‐carers. Although the results in Table A8 should be interpreted with caution as individuals can have substantial proportions of the diary day with no reported time use information. We present an Oaxaca‐Blinder decomposition excluding possible “bad controls” in estimation of the entropy weights in Table A9 and using inverse probability of treatment weights in Table A10. Both sets of results are similar in terms of direction and magnitude when compared to our main results in Table 5.

### 4.5 Decomposition with 11 time use activities

An Oaxaca‐Blinder decomposition with 11 time use categories is presented in Table 6. It reveals that although leisure would have an overall negative effect on carer daily experienced wellbeing there are large competing effects within this activity. For instance, carer daily experienced wellbeing would increase by 0.072 (= 0.035 + 0.032 + 0.005) if they received the higher returns to mass media, sports and outdoor activities and hobbies and computing as non‐carers. These positive effects are dominated by a reduction in daily experienced wellbeing of 0.119 driven by a situation where carers received the same (and therefore lower) wellbeing returns to social life and entertainment activities as non‐carers. Excluding the constant, the overall net effect of time use is close to zero at −0.008 which emphasizes the opposing forces of time use on daily experienced wellbeing. Results in Table 6 are consistent with those in Table 5. An exception is sleep/personal care which is an overall negative contributor compared to the positive contributions in Table 5 of sleep and personal care when included separately. This is because the combined sleep and personal care category has higher returns for carers relative to non‐carers. We present in appendix Table A11 the regressions for carers and non‐carers that are used in the construction of the Oaxaca‐Blinder decomposition presented in Table 6.

**TABLE 6:** Oaxaca‐Blinder decomposition with 11 activity categories

Variable	Time‐composition (SD)	Time effect (SD)	Interaction (SD)	Total (SD)
Time stress: Always	0.009	0.068	−0.010	0.067
	(0.042)	(0.282)	(0.042)	
Time stress: Sometimes	0.007	0.070	−0.011	0.066
	(0.101)	(0.644)	(0.101)	
% day jointly produced	0.003	0.064	−0.003	0.064
	(0.023)	(0.471)	(0.023)	
Mass media	0.010	0.023	0.002	0.035
	(0.039)	(0.392)	(0.039)	
Sports and outdoor activities	0.003	0.023	0.006	0.032
	(0.029)	(0.115)	(0.030)	
Total number of episodes	0.003	0.025	−0.000	0.028
	(0.020)	(1.223)	(0.019)	
Hobbies and computing	−0.001	0.006	−0.000	0.005
	(0.008)	(0.213)	(0.007)	
Study	0.001	0.006	−0.002	0.005
	(0.017)	(0.071)	(0.017)	
Time stress: Missing	−0.007	0.005	0.004	0.002
	(0.118)	(0.176)	(0.118)	
Miscellaneous	0.001	−0.008	−0.001	−0.008
	(0.009)	(0.102)	(0.009)	
Voluntary work and meetings	−0.008	−0.005	0.001	−0.012
	(0.022)	(0.101)	(0.021)	
Travel	−0.001	−0.026	0.001	−0.026
	(0.013)	(0.349)	(0.013)	
Household and family care	0.006	−0.062	−0.002	−0.058
	(0.020)	(0.523)	(0.018)	
Constant		−0.060		−0.060
		(2.711)		
Sleep/Personal care	−0.013	−0.076	0.001	−0.088
	(0.028)	(1.562)	(0.026)	
Social life and entertainment	0.009	−0.122	−0.006	−0.119
	(0.019)	(0.237)	(0.016)	
Total	0.021	−0.068	−0.020	−0.068
	(0.107)	(0.405)	(0.107)	(0.405)

*Note*: The total mean difference is calculated as non‐carer minus carer diaries in line with Equation (1). Diaries that report less than 80% of the day sleeping and with no more than 90 min of incomplete time use and wellbeing information are analyzed. The decomposition is applied to a sample of 5786 non‐carer diaries from 2279 non‐carer individuals and 361 carer diaries from 193 carers. Non‐carers are the reference group. The daily wellbeing score is the average score across the day from a scale coded from 1 (“not at all”) to 7 (“very much”) where higher scores indicate better wellbeing. Time use covariates included in the decomposition are the proportion of the day spent on: sleep/personal care, Study, Household and Family Care, Voluntary work and meetings, Social life and entertainment, Sports and Outdoor activities and Hobbies and computing. Employment is the omitted category. Non‐time use covariates included in the construction of entropy weights are: gender; age; marital status; whether the respondent lives with their partner; the highest education qualification; home ownership; whether the respondent has a long‐term health condition; the number of adults and children in the household; household composition type; region; diary day of the week and diary season. Standard errors clustered at the primary sampling unit in parentheses.

*p < 0.1; **p < 0.05; ***p < 0.01.

### 4.6 Robustness checks

We present the mean difference in daily experienced wellbeing for every 10‐min easing in the completeness of both time use and wellbeing information in appendix Figure A12. The largest difference where non‐carers have higher daily experienced wellbeing than carers is with the sample of complete information on time use and wellbeing across the whole day. This difference switches to non‐carers having lower daily experienced than carers once the restriction on completeness is relaxed until roughly 25 incomplete slots at which point the difference is close to zero. With no restrictions the difference in daily experienced wellbeing is in the same direction as our main analysis sample, although the confidence intervals are large and cross zero across all restrictions.

## 5 DISCUSSION

### 5.1 Main findings

Wellbeing is multifaceted and can be measured in a variety of ways. Measures of evaluative wellbeing and mental health have predominantly been examined in the caregiving literature but there is relatively little evidence that examines daily experienced wellbeing. In this paper, we used information on time use and associated wellbeing to provide evidence on whether there is a daily experienced wellbeing gap by caregiving status and if time use drives any difference. We used the Oaxaca‐Blinder decomposition method combined with entropy weights to address a limitation of this type of decomposition. Our method ensures overlap in terms of socio‐demographic and diary characteristics between carer and non‐carer groups.

Our results show that carers have a higher daily average experienced wellbeing score of 5.76 compared to 5.69 for non‐carers. Interpretation of whether the magnitude of the carer and non‐carer daily average experienced wellbeing difference is economically significant is challenging. However, to provide context around the magnitude of the 0.066 experienced wellbeing difference by caregiver status, it is 26% of the 0.250 higher daily experienced wellbeing found for weekend compared to weekday diaries and more comparable to the effects of longstanding illness status at 0.079 and employment status at 0.089. For both of these differences those with a longstanding illness and those unemployed have the lower wellbeing score. An alternative interpretation of the scale of the wellbeing gap is in terms of the regression coefficients. The wellbeing gap of 0.066 is 5.08 times larger than the non‐carer coefficient for an extra 1% of the day (14.4 min) spent sleeping. Therefore, the wellbeing gap is equivalent to an extra 73 min sleeping, or roughly 15% of the recommended 8 h of sleep.

Decomposition analysis highlights that carers would have even higher daily experienced wellbeing if their time‐composition and time effects were the same as non‐carers. These are driven by carers being more adversely affected in terms of wellbeing than non‐carers by more time stress, increases in the proportion of the day spent jointly producing, increases in the fragmentation of a day and receiving lower wellbeing returns to additional time spent sleeping than non‐carers. The larger of these effects are time stress and sleep which are comparable to the 0.089 difference by employment status.

In contrast, the constant term at −0.254 from the decomposition highlights the unexplained difference in experienced wellbeing from becoming a carer and is the largest contributor to the overall difference in wellbeing. For time use categories, carers would have lower daily experienced wellbeing if they had the same time returns to additional time spent on leisure and non‐market work than non‐carers. However, as carers spend less time on leisure than non‐carers, which ranks as one of the most enjoyable activities, increasing this time use to that of non‐carers would increase daily experienced wellbeing. Analysis from more granular activities highlighted competing effects within leisure.

### 5.2 Comparisons with other studies

Our results for evaluative wellbeing are comparable in terms of direction with the literature on this type of wellbeing where carers report lower life satisfaction than non‐carers (Chen et al., 2019; van den Berg et al., 2014; van den Berg & Ferrer‐I‐Carbonell, 2007). Both Van den Berg et al. (2014) and Leigh (2010) using Australian data find carers had lower life satisfaction, measured on a 1 to 10 scale, by 0.17 for the former and 0.30 for the latter. However, Leigh (2010) restricted carers to those performing at least 10 h of caregiving per week. Chen et al. (2019) found that carers had lower life satisfaction by 0.07 on a four‐point scale using Chinese data. Although the scales and wording may differ between studies, our findings that carers report 0.38 lower overall life satisfaction (on a 7‐point scale) and 0.07 lower life satisfaction nowadays on a 10‐point scale are comparable with the previous studies.

The single‐item experienced wellbeing measures show that carers have lower wellbeing scores than non‐carers which contrasts with our diary derived experienced wellbeing gap. An explanation may be that the diary asks about “enjoyment” where the single‐items ask about “happiness” and “anxiousness”. Carers felt higher levels of worthwhileness than non‐carers which may indicate positive utility derived from the caregiving role (Brouwer et al., 2005). This may be explained by the constant term in the decomposition analysis, which represents additional factors aside from time use, time stress and the characteristics we used to create the entropy weights.

Our work can also be compared to the literature on the experienced wellbeing of the employed and unemployed. Previous work has found differences in evaluative wellbeing between the employed and unemployed but little substantial difference in terms of daily experienced wellbeing (Hoang & Knabe, 2021; Knabe et al., 2010). However, the unemployed are able to compensate for a saddening effect by performing more enjoyable activities such as leisure (Knabe et al., 2010). Informal caregiving for some may be considered a constraint or a constrained choice which may restrict the ability to shift certain uses of time as freely as the unemployed (Al‐Janabi et al., 2018). This relates to how some activities may be time‐bound preventing the ability to reallocate time use, especially among those carers who are also in employment (Hassink & Van den Berg, 2011).

### 5.3 Strengths and limitations

This is the first study to explore experienced daily wellbeing in the context of caregiving using a non‐carer comparator. We make a further contribution to the literature through decomposing experienced wellbeing by various facets of time use and subjective feelings relating to time stress. Previous work by Freedman et al. (2019) has analyzed the experienced wellbeing of carers with a time diary but did not use a non‐carer group.

The findings in this study provide a greater understanding of informal care in relation to a range of wellbeing measures. Instead of obtaining information from five emotions (such as happiness, stress, tiredness, sadness and pain) which must then be aggregated as in the wellbeing module of the American Time Use Survey, the present survey leaves the aggregation of all emotions to the respondent into one number. We have information on experienced wellbeing across 2 days rather than three randomly selected episodes from 1 day in the American Time Use Survey (Stone et al., 2018). Instead of referring to “happiness” or “worthwhileness” the operative emotion in our experienced wellbeing measure is “enjoyment”.

Decomposition analysis has rarely addressed the issue of common support. There is little evidence on how to address the overlap assumption in decomposition analysis beyond the work of Ñopo (2008) and we are the first to our knowledge to apply entropy balancing to this type of analysis. An advantage with entropy balancing is that balance can be achieved across various moments of carer and non‐carer characteristics which is in‐built into the method.

Several limitations of this paper are of note. The Oaxaca‐Blinder decomposition has some drawbacks in terms of the choice of reference category with categorical variables. This issue relates to how contributions of a variable will differ dependent upon the reference category chosen. Although this presents a problem interpreting each variables contribution this does not affect the total time effect and time‐composition components (Jones & Kelley, 1984). Alternative decompositions use a twofold approach which would combine the interaction effect with the time effect component and use coefficients based on a combination of the two groups as a benchmark of comparison. The threefold decomposition provides extra detail through separation of interaction effect from both the time‐composition and time effect components. We found that the interaction effect was in most cases smaller than the time effect component but only in a few cases larger than the time‐composition effect. Standard errors were large for all variables in the decomposition which may in part be due to the small number of carer diaries at 361 (from 206 carers), however, standard errors in Oaxaca‐Blinder decompositions are often interpreted with caution (Etezady et al., 2021).

A further limitation of the study are possible unobservable characteristics that are correlated with time use and wellbeing producing bias in our time use coefficients. The constant term represents a large proportion of the time effects component and can be considered to include both unobserved time‐composition and effect variables. Whilst unobservables pose a potential issue in this study, means to address this by using panel data may not be feasible and should be balanced against the rich information available in the UK Time Use Survey. A related issue is that this study does not account for what time is replaced (or added) if carers were to increase (or decrease) their time spent on a particular activity to bring in line with non‐carers. Application of entropy weights aims to ensure that the non‐carer group are similar to the carer group in terms of observable characteristics, therefore, the opportunity costs of changes in time use should also be comparable across groups.

It was not entirely clear what sample restrictions should be placed on the time diaries with regards to the number of incomplete time slots and extremes in certain uses of time. We explored results from several restrictions on the completeness of time use and wellbeing information and found the contributions of most time uses to be broadly consistent across these restrictions. The difference ranged across each sample restriction from carers having lower wellbeing by 0.042 from the sample with complete time use and wellbeing slots and higher wellbeing by 0.066 (on a scale of 0–7) with the main analysis sample relative to non‐carers. Although the confidence intervals of these differences were large and crossed zero. The characteristics of carers at both extremes of the time and wellbeing sample restrictions were similar easing generalizability concerns with select samples.

### 5.4 Future work

We used a detailed snapshot of time use and experienced wellbeing. Future research could collect time use, experienced and evaluative wellbeing over time to form a panel. A decomposition of changes in these measures over time and prior to caregiving may help understand if time use is related to selection into caregiving and better identify a caregiving spell. This may help understand if experienced wellbeing varies in line with temporary changes and whether there is a degree of adaptation to long‐term circumstances such as with long term health conditions. It may also provide insight into whether the accumulation of jointly produced activity and a more fragmented day over a caregiving spell manifests in reductions in evaluative wellbeing. Similarly, recording of positive and negative emotions as with the daily reconstruction method would yield additional insight beyond the internal aggregation made by respondents in the 2014/15 UK Time Use Survey.

### 5.5 Implications

Several studies have shown that informal carers have lower life satisfaction than non‐carers, however, we show this is not the case for our measure of experienced wellbeing. We provide an explanation for the absence of a substantial experienced wellbeing gap between carers and non‐carers by demonstrating that this is the result of offsetting time‐composition and saddening effects. In other words, carers use their time differently and receive different returns to time use than non‐carers, but in aggregate these offset to a small but positive experienced wellbeing difference for carers. Our results provide an insight into what aspects of carers' day‐to‐day lives, if altered to become identical to that of non‐carers, would increase or decrease experienced wellbeing. For example, the lower wellbeing returns that carers experience for sleep may be indicative of the quality of that sleep, as previous evidence has highlighted that carers experience interrupted sleep (Sacco et al., 2018).

Dolan et al. (2017) and Dolan and Metcalfe (2012) argue that a variety of wellbeing measures should be used to draw conclusions about individuals' lives. We carry this argument to caregiving and emphasize that the wellbeing effects of informal caregiving are complex and varied.

## CONFLICT OF INTEREST

None of the authors have a conflict of interest with respect to this manuscript.

## Supporting information

Supporting Information S1

## Data Availability

The study uses data from the 2014/15 UK Time Use Survey (UKTUS) which is available by application from the UK Data Service (https://ukdataservice.ac.uk/). The authors are not permitted to release the copy of the UKTUS used for the study.
