# Supplementary material for: Informal caregiving, time use and experienced wellbeing
Source: Health Econ. 2022 Oct 27;32(2):356–74. doi: 10.1002/hec.4624 (PMC10092671; doi:10.1002/hec.4624)
Supplement: Supplementary file 1 — Supporting Information S1 [file HEC-32-356-s001.docx]

# Appendix

| Table A1: Two-digit activities applied to six activity groups | |
| --- | --- |
| Author derived activity grouping | Two-digit time use survey activities |
| Sleep |  |
|  | Sleep |
| Personal care |  |
|  | Eating, Other personal care |
|  |  |
| Market work |  |
|  | Main job, Second job, Activities related to employment, School or university, Free time study, Travel – work, Travel - study |
| Non-market work |  |
|  | Food management, Household upkeep, Making and care for textiles, Gardening and pet care, Construction and repairs, Shopping and services, Household management, Childcare of own household members, Help to an adult household member, Organisational work, Informal help to other households, Participatory activities, Travel – domestic, Travel – voluntary civic |
| Leisure |  |
|  | Social life, Entertainment and culture, Resting – Time out, Physical exercise, Productive exercise, Sports related activities, Arts and hobbies, Computing, Games, Reading, TV and video, Radio and music, Travel – social/entertainment, Travel – sport/exercises, Travel – hobbies/games |
| Uncategorised |  |
|  | Punctuating activity/ fill in diary time/ other specified or unspecified time, Travel - other |

| Table A2: Two-digit activities applied to eleven activity groups | |
| --- | --- |
| Author derived activity grouping | Two-digit time use survey activities |
| Sleep/Personal Care |  |
|  | Sleep, eating, other personal care |
| Employment |  |
|  | Main job, Second job, Activities related to employment |
| Study |  |
|  | School or university, Free time study |
| Household and Family Care |  |
|  | Food management, Household upkeep, Making and care for textiles, Gardening and pet care, Construction and repairs, Shopping and services, Household management, Childcare, Help to an adult household member |
| Voluntary work and meetings |  |
|  | Organisational work, Informal help to other households, Participatory activities |
| Social life and entertainment |  |
|  | Social life and entertainment, entertainment and culture, resting/time out |
| Sports and outdoor activities |  |
|  | Physical exercise, ball games, other physical exercise, productive exercise |
| Hobbies and Computing |  |
|  | Arts and hobbies, hobbies, computing, games |
| Mass media |  |
|  | Reading, tv/video and dvd, radios and recordings, |
| Miscellaneous |  |
|  | Punctuating activity/ fill in diary time/ other specified or unspecified time |
| Travel |  |
|  | Travel – work, Travel – study, Travel – domestic, Travel – voluntary civic, Travel – social/entertainment, Travel – sport/exercises, Travel – hobbies/games, Travel - other |

| Table A3: Description of the variables used for entropy balancing | |
| --- | --- |
| Variable | Description |
| Female | 0 if no; 1 if yes |
| Age | Number of years old |
| Marital status: Single/Never Married | 0 if no; 1 if yes |
| Marital status: Married/cohabiting | 0 if no; 1 if yes |
| Marital status: Divorced/Widowed | 0 if no; 1 if yes |
| Marital status: Missing | 0 if no; 1 if yes |
| Lives with partner | 0 if no; 1 if yes |
| Highest education qualification: University or higher | 0 if no; 1 if yes |
| Highest education qualification: Higher secondary | 0 if no; 1 if yes |
| Highest education qualification: Secondary | 0 if no; 1 if yes |
| Highest education qualification: Other | 0 if no; 1 if yes |
| Highest education qualification: Missing | 0 if no; 1 if yes |
| Owns residing home | 0 if no; 1 if yes |
| Health condition: No long-term health condition | 0 if no; 1 if yes |
| Health condition: Has a long-term health condition | 0 if no; 1 if yes |
| Health condition: Missing | 0 if no; 1 if yes |
| # of adults in the HH | Number of adults residing in the Household |
| # aged 0-4 years old in the HH | Number of children between 0-4 years old residing in the Household |
| # aged 5-15 years old in the HH | Number of children between 5-15 years old residing in the Household |
| HH type: Single person | 0 if no; 1 if yes |
| HH type: Married/Cohabiting with children | 0 if no; 1 if yes |
| HH type: Married/Cohabiting no children | 0 if no; 1 if yes |
| HH type: Single parent with children | 0 if no; 1 if yes |
| HH type: Single parent no children | 0 if no; 1 if yes |
| HH type: Unclassified – married/cohabiting | 0 if no; 1 if yes |
| HH type: Unclassified – single parent | 0 if no; 1 if yes |
| HH type: Other households | 0 if no; 1 if yes |
| Region: London & the south east of England | 0 if no; 1 if yes |
| Region: Rest of England | 0 if no; 1 if yes |
| Region: Wales | 0 if no; 1 if yes |
| Region: Scotland | 0 if no; 1 if yes |
| Season: Spring | 0 if no; 1 if yes |
| Season: Summer | 0 if no; 1 if yes |
| Season: Autumn | 0 if no; 1 if yes |
| Season: Winter | 0 if no; 1 if yes |
| Day of the week: Monday | 0 if no; 1 if yes |
| Day of the week: Tuesday | 0 if no; 1 if yes |
| Day of the week: Wednesday | 0 if no; 1 if yes |
| Day of the week: Thursday | 0 if no; 1 if yes |
| Day of the week: Friday | 0 if no; 1 if yes |
| Day of the week: Saturday | 0 if no; 1 if yes |
| Day of the week: Sunday | 0 if no; 1 if yes |

Table A4: Characteristics of carers and non-carers across sample restrictions

|  | Diaries with complete time and wellbeing slots | | Diaries with any incomplete time and wellbeing slots | |
| --- | --- | --- | --- | --- |
| Covariate | Non-carer | Carer | Non-carer | Carer |
|  | (4016 diaries) | (246 diaries) | (8595 diaries) | (546 diaries) |
|  | Mean (SD) or % | | | |
| **Individual level covariates:** |  |  |  |  |
| Female | 0.50 | 0.60 | 0.51 | 0.59 |
| Age (years) | 48.62 (18.30) | 52.35 (16.43) | 48.72 (18.29) | 52.01 (16.71) |
| Marital status: Single/Never Married | 0.18 | 0.13 | 0.18 | 0.15 |
| Marital status: Married/ cohabiting | 0.58 | 0.78 | 0.58 | 0.77 |
| Marital status: Divorced/Widowed | 0.16 | 0.10 | 0.16 | 0.08 |
| Marital status: missing | 0.08 | 0.00 | 0.08 | 0.00 |
| Lives with partner | 0.64 | 0.79 | 0.65 | 0.77 |
| Education: University or Higher | 0.38 | 0.35 | 0.39 | 0.33 |
| Education Higher secondary qualification | 0.18 | 0.15 | 0.17 | 0.16 |
| Education: Secondary qualification | 0.23 | 0.27 | 0.23 | 0.30 |
| Education: Other qualification | 0.11 | 0.17 | 0.10 | 0.16 |
| Education: Missing | 0.10 | 0.07 | 0.10 | 0.05 |
| Tenure: Owns home | 0.71 | 0.61 | 0.71 | 0.62 |
| Born: Not UK | 0.12 | 0.09 | 0.12 | 0.10 |
| Born: UK | 0.80 | 0.91 | 0.79 | 0.90 |
| Born: Missing | 0.08 | 0.00 | 0.08 | 0.00 |
| Long standing health condition: No | 0.58 | 0.50 | 0.58 | 0.48 |
| Long standing health condition: Yes | 0.34 | 0.50 | 0.34 | 0.51 |
| Long standing health condition: missing | 0.08 | 0.00 | 0.08 | 0.01 |
| Number of Adults in HH | 2.26 (1.08) | 2.64 (1.10) | 2.29 (1.11) | 2.63 (1.08) |
| Number aged 11-15 years old in HH | 0.15 (0.42) | 0.24 (0.51) | 0.15 (0.43) | 0.21 (0.50) |
| Number aged 5-10 years old in HH | 0.18 (0.49) | 0.32 (0.66) | 0.18 (0.49) | 0.28 (0.63) |
| Number aged 0-4 years old in HH | 0.19 (0.50) | 0.11 (0.38) | 0.19 (0.49) | 0.15 (0.45) |
| Region: London and the South East | 0.32 | 0.25 | 0.32 | 0.25 |
| Region: The rest of England | 0.67 | 0.73 | 0.67 | 0.74 |
| Region: Wales | 0.01 | 0.02 | 0.01 | 0.01 |
| Region: Scotland | 0.00 | 0.01 | 0.00 | 0.00 |
| HH type: Single person | 0.17 | 0.00 | 0.16 | 0.00 |
| HH type: Married/Cohab with children | 0.18 | 0.17 | 0.18 | 0.15 |
| HH type: Married/Cohab no children | 0.30 | 0.33 | 0.31 | 0.36 |
| HH type: Single parent with children | 0.03 | 0.05 | 0.03 | 0.04 |
| HH type: Single parent no children | 0.03 | 0.04 | 0.04 | 0.08 |
| HH type: Unclassified – married/cohab | 0.16 | 0.21 | 0.16 | 0.21 |
| HH type: Unclassified – single parent | 0.08 | 0.15 | 0.08 | 0.12 |
| HH type: Other households | 0.04 | 0.05 | 0.04 | 0.05 |
| **Diary level covariates:** |  |  |  |  |
| Season: Spring | 0.21 | 0.23 | 0.21 | 0.23 |
| Season: Summer | 0.18 | 0.25 | 0.18 | 0.25 |
| Season: Autumn | 0.34 | 0.29 | 0.34 | 0.29 |
| Season: Winter | 0.28 | 0.23 | 0.28 | 0.23 |
| Day of the week: Monday | 0.25 | 0.24 | 0.25 | 0.24 |
| Day of the week: Tuesday | 0.09 | 0.10 | 0.09 | 0.10 |
| Day of the week: Wednesday  Wednesday | 0.10 | 0.10 | 0.10 | 0.10 |
| Day of the week: Thursday | 0.09 | 0.11 | 0.09 | 0.11 |
| Day of the week: Friday | 0.10 | 0.10 | 0.10 | 0.10 |
| Day of the week: Saturday | 0.11 | 0.09 | 0.11 | 0.09 |
| Day of the week: Sunday | 0.25 | 0.26 | 0.25 | 0.26 |
| *Notes:* Diaries with more than 80% of the day spent on sleep are excluded from each sample | | | | |

Figure A5: Weighted linear trend between daily experienced wellbeing and the proportion of the day spent on six activities


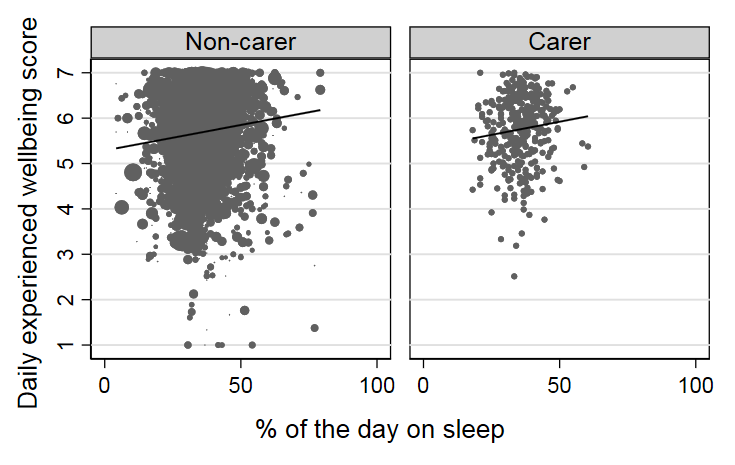


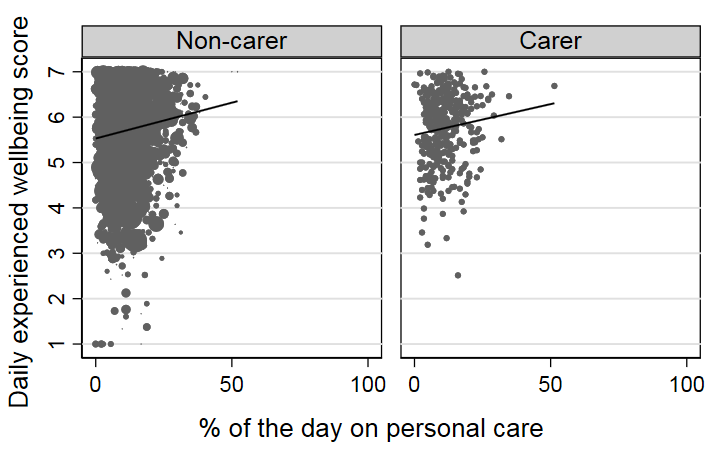


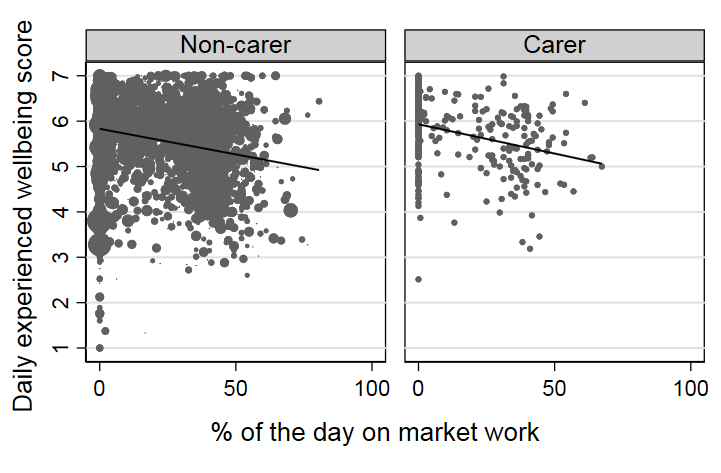


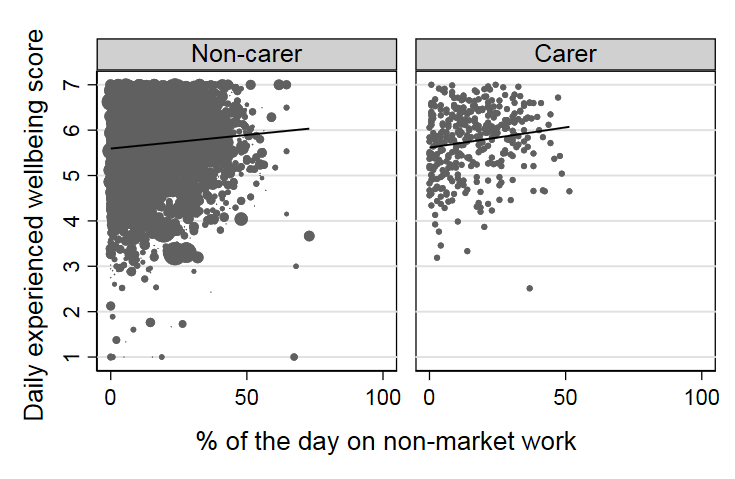


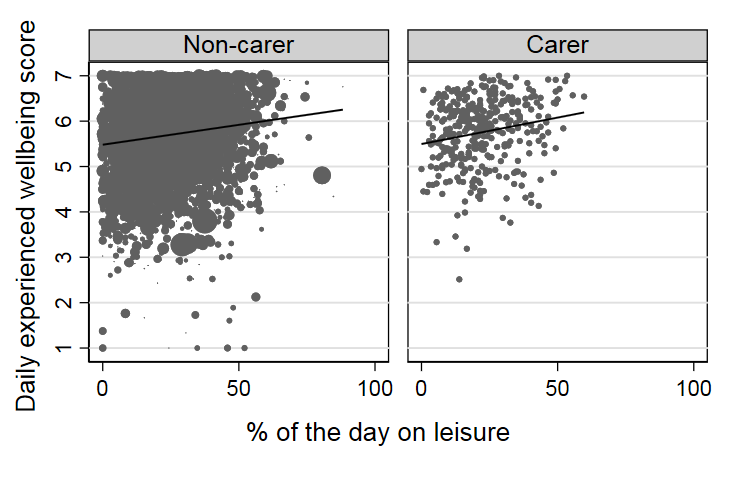


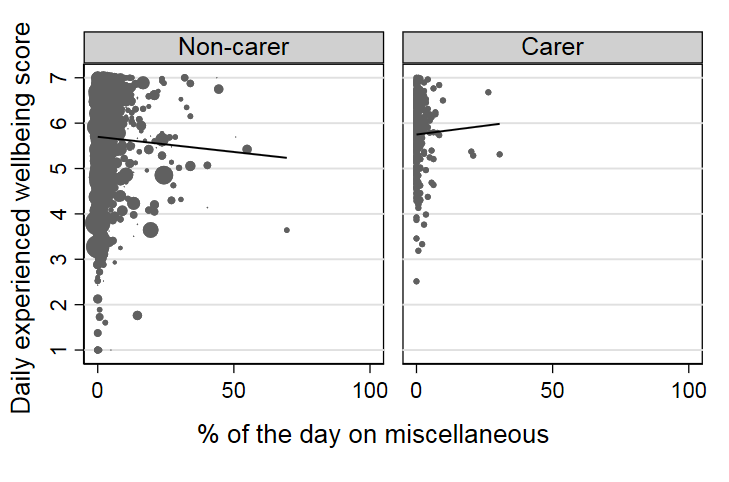


*Notes:* Entropy weights have been applied. The black line indicates the linear trend and each grey dot indicates an observation. Diaries that report less than 80% of the day sleeping and with no more than 90 minutes of incomplete time use and wellbeing information are analysed

| Table A6: Regressions on daily experienced wellbeing stratified by caregiving status across two samples | | | | |
| --- | --- | --- | --- | --- |
|  | Excluding Diaries with more than 80% of the day spent sleeping | | | |
|  | Diaries with complete time and wellbeing slots | | Diaries with no restrictions on the completeness of time and wellbeing slots | |
|  | Carers | Non-carers | Carers | Non-carers |
| Sleep | 0.008 | 0.013*** | 0.016*** | 0.012*** |
|  | (0.006) | (0.003) | (0.005) | (0.002) |
| Personal care | 0.020** | 0.025*** | 0.015** | 0.017*** |
|  | (0.008) | (0.005) | (0.007) | (0.003) |
| Non-market | 0.010* | 0.010*** | 0.012*** | 0.012*** |
|  | (0.005) | (0.003) | (0.004) | (0.002) |
| Leisure | 0.015*** | 0.012*** | 0.014*** | 0.014*** |
|  | (0.004) | (0.002) | (0.003) | (0.002) |
| Miscellaneous | 0.006 | 0.000 | 0.010 | 0.010** |
|  | (0.014) | (0.006) | (0.014) | (0.004) |
| % Jointly produced | -0.232 | -0.183 | -0.076 | -0.176 |
|  | (0.338) | (0.224) | (0.267) | (0.176) |
| Total number of episodes | -0.004 | -0.007*** | -0.006* | -0.005*** |
|  | (0.005) | (0.002) | (0.004) | (0.002) |
| Time stress: Always | -0.325 | 0.018 | -0.240 | -0.002 |
|  | (0.212) | (0.103) | (0.161) | (0.074) |
| Time stress: Sometimes | -0.175 | 0.000 | -0.012 | -0.001 |
|  | (0.162) | (0.087) | (0.130) | (0.064) |
| Time stress: Missing | -0.238 | -0.042 | -0.040 | -0.065 |
|  | (0.206) | (0.133) | (0.159) | (0.089) |
| Constant | 5.681*** | 5.722*** | 5.697*** | 5.630*** |
|  | (0.057) | (0.031) | (0.049) | (0.025) |
| Diaries | 246 | 4016 | 546 | 8595 |
| Individuals | 156 | 1915 | 250 | 2853 |

*Notes*: The daily wellbeing score is average score across the day from a scale coded from 1 (‘not at all’) to 7 (‘very much’) where higher scores indicate better wellbeing. Entropy weights have been applied to each regression. The omitted time category and time stress category is employment and always, respectively. Non-time related covariates included in the construction of entropy weights are: age, gender, education, marital status, number of children in the household, number of adults in the household, an indicator for the presence of a long-standing illness, whether the respondent was born in the UK, diary day of the week and diary season. Standard errors clustered at the primary sampling unit in parentheses: *p<0.1; **p<0.05; ***p<0.01

Table A7: Oaxaca-Blinder decomposition on diaries with complete time and wellbeing slots

| Variable | Time-Composition  (SD) | Time Effect  (SD) | Interaction  (SD) | Total  (SD) |
| --- | --- | --- | --- | --- |
| Sleep | 0.002 | 0.160 | 0.001 | 0.163 |
|  | (0.011) | (1.912) | (0.011) |  |
| Time stress: Sometimes | 0.009 | 0.093 | -0.009 | 0.093 |
|  | (0.074) | (0.728) | (0.074) |  |
| Time stress: Always | 0.012 | 0.065 | -0.013 | 0.064 |
|  | (0.068) | (0.342) | (0.068) |  |
| Personal Care | -0.019 | 0.049 | -0.004 | 0.026 |
|  | (0.067) | (0.788) | (0.066) |  |
| Time stress: Missing | -0.015 | 0.022 | 0.012 | 0.019 |
|  | (0.108) | (0.199) | (0.107) |  |
| % day Jointly produced | 0.003 | 0.010 | -0.001 | 0.012 |
|  | (0.032) | (0.566) | (0.032) |  |
| Non-Market | 0.000 | 0.009 | 0.000 | 0.009 |
|  | (0.010) | (0.675) | (0.002) |  |
| Miscellaneous | 0.001 | -0.005 | -0.001 | -0.005 |
|  | (0.016) | (0.094) | (0.016) |  |
| Leisure | 0.035 | -0.068 | -0.007 | -0.040 |
|  | (0.087) | (0.796) | (0.085) |  |
| Total number of episodes | 0.005 | -0.087 | 0.003 | -0.079 |
|  | (0.052) | (1.380) | (0.051) |  |
| Constant |  | -0.223 |  | -0.223 |
|  |  | (3.453) |  |  |
| Total | 0.034 | 0.027 | -0.019 | 0.041 |
|  | (0.159) | (0.486) | (0.158) | (0.486) |
| *Notes:* The total mean difference is calculated as non-carer minus carer diaries in line with equation 1. Diaries that report less than 80% of the day sleeping and with no incomplete time use and wellbeing slots are analysed. The decomposition is applied to a sample of 4016 non-carer diaries from 1915 non-carer individuals and 246 carer diaries from 156 carers. The daily wellbeing score is the average score across the day from a scale coded from 1 (‘not at all’) to 7 (‘very much’) where higher scores indicate better wellbeing. Entropy weights have been applied to each regression. The omitted time category and time stress category is employment and always, respectively. Non-time related covariates included in the construction of entropy weights are: age, gender, education, marital status, number of children in the household, number of adults in the household, an indicator for the presence of a long-standing illness, whether the respondent was born in the UK, diary day of the week and diary season. standard errors clustered at the primary sampling unit in parentheses: *p<0.1; **p<0.05; ***p<0.01 | | | | |

Table A8: Oaxaca-Blinder decomposition on diaries with no restrictions on the completeness of time use and wellbeing slots

| Variable | Time-Composition (SD) | Time Effect (SD) | Interaction (SD) | Total  (SD) |
| --- | --- | --- | --- | --- |
| Time stress: Always | 0.011 | 0.045 | -0.010 | 0.046 |
|  | (0.059) | (0.249) | (0.059) |  |
| Total number of episodes | -0.001 | 0.017 | 0.000 | 0.016 |
|  | (0.008) | (1.078) | (0.007) |  |
| Personal Care | -0.007 | 0.022 | -0.001 | 0.014 |
|  | (0.026) | (0.602) | (0.025) |  |
| Constant |  | 0.008 |  | 0.008 |
|  |  | (2.441) |  |  |
| Time stress: Sometimes | 0.001 | 0.006 | -0.001 | 0.006 |
|  | (0.073) | (0.584) | (0.073) |  |
| Leisure | 0.008 | -0.005 | -0.000 | 0.003 |
|  | (0.018) | (0.598) | (0.015) |  |
| Miscellaneous | 0.002 | 0.001 | 0.000 | 0.003 |
|  | (0.025) | (0.235) | (0.025) |  |
| Time stress: Missing | -0.003 | -0.003 | -0.002 | -0.008 |
|  | (0.096) | (0.164) | (0.096) |  |
| Non-Market | -0.007 | -0.007 | 0.000 | -0.014 |
|  | (0.021) | (0.527) | (0.019) |  |
| % day Jointly produced | 0.001 | -0.018 | 0.001 | -0.016 |
|  | (0.025) | (0.400) | (0.025) |  |
| Sleep | 0.002 | -0.125 | -0.001 | -0.124 |
|  | (0.009) | (1.474) | (0.007) |  |
| Total | 0.007 | -0.060 | -0.013 | -0.066 |
|  | (0.108) | (0.403) | (0.107) | (0.403) |
| *Notes:* Diaries that report less than 80% of the day sleeping and with any amount of incomplete time use and wellbeing information are analysed. The decomposition is applied to a sample of 8595 non-carer diaries from 2853 non-carer individuals and 546 carer diaries from 250 carers. The daily wellbeing score is the average score across the day from a scale coded from 1 (‘not at all’) to 7 (‘very much’) where higher scores indicate better wellbeing. Entropy weights have been applied to each regression. The omitted time category and time stress category is employment and always, respectively. Non-time related covariates included in the construction of entropy weights are: age, gender, education, marital status, number of children in the household, number of adults in the household, an indicator for the presence of a long-standing illness, whether the respondent was born in the UK, diary day of the week and diary season. standard errors clustered at the primary sampling unit in parentheses: *p<0.1; **p<0.05; ***p<0.01 | | | | |

Table A9: Oaxaca-Blinder decomposition without possible ‘bad controls’ included in entropy weight estimation

| Variable | Time-Composition (SD) | Time Effect (SD) | Interaction (SD) | Total  (SD) |
| --- | --- | --- | --- | --- |
| Time stressed: Sometimes | 0.011 | 0.082 | -0.014 | 0.079 |
|  | (0.111) | (0.651) | (0.111) |  |
| Time stressed: Always | 0.006 | 0.059 | -0.006 | 0.059 |
|  | (0.028) | (0.259) | (0.028) |  |
| % Jointly produced | 0.002 | 0.057 | -0.003 | 0.056 |
|  | (0.022) | (0.451) | (0.022) |  |
| Sleep | 0.002 | 0.036 | 0.000 | 0.038 |
|  | (0.009) | (1.589) | (0.008) |  |
| Total number of episodes | 0.004 | 0.013 | 0 | 0.017 |
|  | (0.020) | (1.155) | (0.019) |  |
| Time stressed: Missing | -0.011 | 0.006 | 0.005 | 0.000 |
|  | (0.125) | (0.165) | (0.125) |  |
| Personal care | -0.015 | 0.008 | -0.001 | -0.008 |
|  | (0.050) | (0.641) | (0.049) |  |
| Miscellaneous | 0.001 | -0.013 | -0.001 | -0.013 |
|  | (0.008) | (0.102) | (0.008) |  |
| Leisure | 0.024 | -0.071 | -0.005 | -0.052 |
|  | (0.052) | (0.669) | (0.051) |  |
| Non-Market | 0.001 | -0.06 | 0.000 | -0.059 |
|  | (0.012) | (0.576) | (0.004) |  |
| Constant |  | -0.154 |  | -0.154 |
|  |  | (2.684) |  |  |
| Total | 0.026 | -0.038 | -0.025 | -0.037 |
|  | (0.126) | (0.413) | (0.125) | (0.413) |
| *Notes:* The total mean difference is calculated as non-carer minus carer diaries in line with equation 1. Diaries that report less than 80% of the day sleeping and with no more than 90 minutes of incomplete time use and wellbeing information are analysed. The decomposition is applied to a sample of 5786 non-carer diaries from 3304 non-carer individuals and 361 carer diaries from 206 carers. Non-carers are the reference group. The daily experienced wellbeing score is the average score across the day from a scale coded from 1 (‘not at all’) to 7 (‘very much’) where higher scores indicate better wellbeing. Entropy weights have been applied to the decomposition. The omitted time category and time stress category is employment and always, respectively. Non-time related covariates included in the construction of entropy weights are: age; gender; education; number of children in the household; number of adults in the household; whether the respondent was born in the UK; diary day of the week; and diary season. Standard errors clustered at the primary sampling unit in parentheses: *p<0.1; **p<0.05; ***p<0.01 | | | | |

Table A10: Oaxaca-Blinder decomposition with inverse probability weights

| Variable | Time-Composition (SD) | Time Effect (SD) | Interaction (SD) | Total  (SD) |
| --- | --- | --- | --- | --- |
| Sleep | 0.001 | 0.082 | 0.000 | 0.083 |
|  | (0.005) | (0.221) | (0.001) |  |
| Time stressed: Sometimes | 0.012 | 0.083 | -0.014 | 0.081 |
|  | (0.014) | (0.086) | (0.016) |  |
| Time stressed: Always | 0.009 | 0.063 | -0.01 | 0.062 |
|  | (0.011) | (0.035) | (0.012) |  |
| % Jointly produced | 0.002 | 0.044 | -0.002 | 0.044 |
|  | (0.004) | (0.068) | (0.004) |  |
| Total number of episodes | 0.004 | 0.032 | -0.001 | 0.035 |
|  | (0.007) | (0.159) | (0.003) |  |
| Personal care | -0.016 | 0.026 | -0.002 | 0.008 |
|  | (0.009) | (0.090) | (0.007) |  |
| Time stressed: missing | -0.01 | 0.005 | 0.004 | -0.001 |
|  | (0.014) | (0.024) | (0.017) |  |
| Miscellaneous | 0.002 | -0.012 | -0.001 | -0.011 |
|  | (0.003) | (0.016) | (0.003) |  |
| Leisure | 0.022 | -0.059 | -0.004 | -0.041 |
|  | (0.012) | (0.092) | (0.007) |  |
| Non-market work | -0.001 | -0.056 | 0.000 | -0.057 |
|  | (0.012) | (0.080) | (0.003) |  |
| Constant |  | -0.259 |  | -0.259 |
|  |  | (0.361) |  |  |
| Total | 0.026 | -0.050 | -0.030 | -0.054 |
|  | (0.023) | (0.055) | (0.021) | (0.059) |
| *Notes:* The total mean difference is calculated as non-carer minus carer diaries in line with equation 1. Diaries that report less than 80% of the day sleeping and with no more than 90 minutes of incomplete time use and wellbeing information are analysed. The decomposition is applied to a sample of 5303 non-carer diaries from 2131 non-carer individuals and 361 carer diaries from 206 carers. Non-carers are the reference group. The daily wellbeing score is the average score across the day from a scale coded from 1 (‘not at all’) to 7 (‘very much’) where higher scores indicate better wellbeing. Entropy weights have been applied to each regression. The omitted time category and time stress category is employment and always, respectively. Non-time related covariates included in the construction of entropy weights are: age, gender, education, marital status, number of children in the household, number of adults in the household, an indicator for the presence of a long-standing illness, whether the respondent was born in the UK, diary day of the week and diary season. standard errors clustered at the primary sampling unit in parentheses: *p<0.1; **p<0.05; ***p<0.01 | | | | |

Table A11: Regressions on daily experienced wellbeing stratified by caregiving status

|  | Daily Experienced Wellbeing | |
| --- | --- | --- |
|  | Carers | Non-Carers |
| Sleep/Personal care | 0.017*** | 0.015*** |
|  | (0.004) | (0.002) |
| Study | -0.002 | 0.004 |
|  | (0.007) | (0.004) |
| Household and Family Care | 0.015*** | 0.010*** |
|  | (0.005) | (0.004) |
| Voluntary work and meetings | 0.031*** | 0.027*** |
|  | (0.009) | (0.005) |
| Social life and entertainment | 0.031*** | 0.010** |
|  | (0.005) | (0.005) |
| Sports and Outdoor activities | 0.010 | 0.031*** |
|  | (0.013) | (0.004) |
| Hobbies and computing | 0.012 | 0.014*** |
|  | (0.010) | (0.004) |
| Mass Media | 0.009** | 0.011*** |
|  | (0.004) | (0.002) |
| Travel | 0.006 | 0.001 |
|  | (0.008) | (0.004) |
| Miscellaneous | 0.008 | 0.001 |
|  | (0.011) | (0.011) |
| % Jointly produced | -0.289 | 0.049 |
|  | (0.295) | (0.230) |
| Total number of episodes | -0.006 | -0.005** |
|  | (0.004) | (0.002) |
| Time stress: Sometimes | -0.382* | 0.017 |
|  | (0.201) | (0.092) |
| Time stress: Never | -0.081 | 0.043 |
|  | (0.137) | (0.081) |
| Time stress: Missing | -0.101 | -0.051 |
|  | (0.192) | (0.129) |
| Constant | 5.758*** | 5.692*** |
|  | (0.048) | (0.031) |
| Diaries | 361 | 5786 |
| Individuals | 193 | 2279 |
| *Notes*: Diaries that report less than 80% of the day sleeping and with no more than 90 minutes of incomplete time use and wellbeing information are analysed. The daily wellbeing score is average score across the day from a scale coded from 1 (‘not at all’) to 7 (‘very much’) where higher scores indicate better wellbeing. Entropy weights have been applied to each regression. The omitted time category and time stress category is employment and always, respectively. Non-time related covariates included in the construction of entropy weights are: age, gender, education, marital status, number of children in the household, number of adults in the household, an indicator for the presence of a long-standing illness, whether the respondent was born in the UK, diary day of the week and diary season. Standard errors clustered at the primary sampling unit in parentheses: *p<0.1; **p<0.05; ***p<0.01 | | |

Figure A12: Difference in mean experienced wellbeing scores between carers and non-carer by the completeness of the time diary


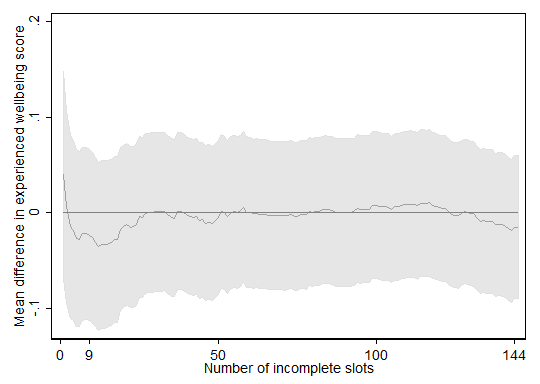


*Notes:* The difference in experienced wellbeing is the non-carers wellbeing score minus the carer wellbeing score. The light grey line indicates the mean difference, the black line indicates the 90 minutes sample restriction and the shaded grey area indicates the 95% confidence interval around the difference. All differences exclude diaries that report over 80% of the day on sleep. Entropy weights are not applied to each easing of restrictions.
